# A Low-Cost pH Sensor for Real-Time Monitoring of Aquaculture Systems in a Multi-Layer Wireless Sensor Network

**DOI:** 10.3390/s25092824

**Published:** 2025-04-30

**Authors:** Binta Mohammed Adib Zeta, Sifat U. Alam, Gazi M. A. Ehsan Ur Rahman, Khawza Iftekhar Uddin Ahmed

**Affiliations:** 1 Department of Electrical and Electronic Engineering, United International University, Dhaka 1212, Bangladesh; bzeta173013@bseee.uiu.ac.bd (B.M.A.Z.); salam171048@bseee.uiu.ac.bd (S.U.A.); erahman.uiu@gmail.com (G.M.A.E.U.R.); 2 Department of Electrical and Electronic Engineering, Green University of Bangladesh, Narayanganj 1461, Bangladesh

**Keywords:** aquaculture, pH sensor, electrode, temperature compensation

## Abstract

For aquaculture systems, pH is the prime quality indicator and is highly related to other water quality indicators like ammonia and ammonium ions. The available pH sensors using chemical references are not suitable for continuous in situ monitoring of aquaculture systems due to their frequent calibration requirement and high cost. This research develops a pH sensor with temperature compensation implementing a machine learning (ML) algorithm. Unlike traditional methods, this sensor utilizes electronic calibration, eliminating the need for chemical calibration and ongoing maintenance efforts. The application of this low-cost sensor is particularly well suited for in situ aquaculture scenarios, where multiple local sensor nodes operate under the control of a master node. The test results of the developed sensor show an improved sensitivity from 0.288 µA/pH to 0.316 µA/pH compared to the available pH sensors. Additionally, the response time has been improved from 1 s to 125 ms, showcasing the suitability of this pH sensor for real-time water quality monitoring of aquaculture applications.

## 1. Introduction

**Figure 1:**
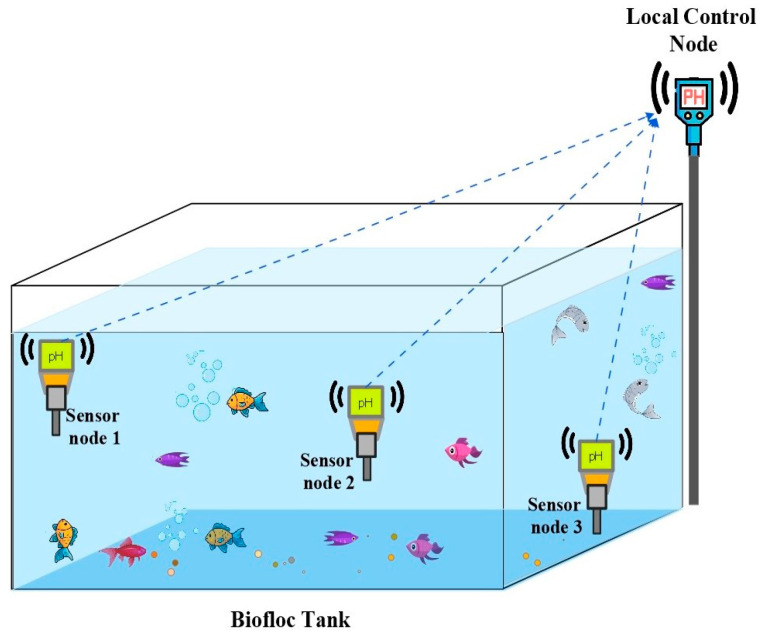
Multiple sensors in a Biofloc tank at different depth and position communicating with a local control node.

**Figure 2:**
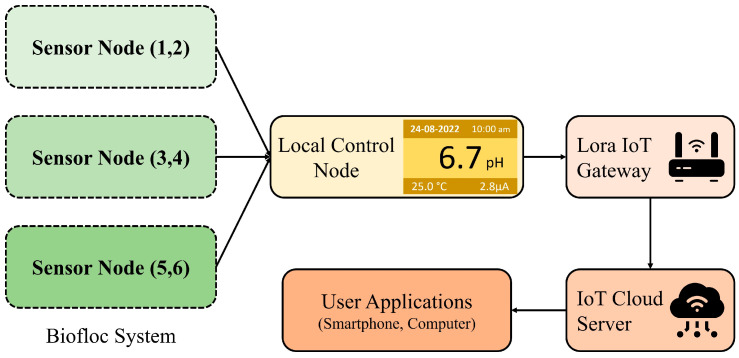
System architecture with multi depth sensor nodes and control node, along with WSN-GW-IoT-User application.

Because of the increasing global population, we need environmentally friendly methods for food production. Biofloc fish farming provides a reliable and eco-friendly solution, and has become popular among fish farmers [1]. The success of fish production in aquaculture systems depends on water quality, which includes pH, ammonia, dissolved oxygen (DO), total dissolved solids (TDS), and chloride [2]. To keep fish healthy in a Biofloc tank, it’s crucial to monitor these factors consistently. However, many available sensors require costly equipment and manual operation, and sometimes they provide inaccurate readings due to calibration issues [3]. Therefore, we aim to create cost-effective IoT sensors for real-time monitoring of the key water quality parameters. Integrating existing sensors in an IoT sensor node posed challenges due to compatibility issues. To overcome this, we have developed a new pH sensor that seamlessly interfaces with the real-time IoT sensor node, complete with temperature compensation. pH stands out as a crucial water quality indicator, reflecting various other parameters, such as alkalinity, CO_2_, dissolved oxygen (DO), total dissolved solids (TDS), biochemical oxygen demand (BOD), chloride, ammonia, and ammonium ions in aquatic environments [4]. Additionally, pH plays a role in solubility reactions, metal concentration, and chemical processes such as oxidation and reduction [5]. Figure 1 outlines the proposed setup for an aquaculture system, where sensors are placed at different depths within a Biofloc tank for effective data collection. Through the sensor nodes at different depths, the control node obtains pH data from the tank. After that, the multipoint data are compressed and processed at the control node. Figure 2 presents the potential architecture of a smart aquaculture system. Sensor nodes are connected to a local control node wirelessly through LoRa links, which serves as the concentrator. All the sensor nodes form a local Wireless Sensor Network (WSN) with the concentrator. The concentrator collects data from the sensor nodes and transmits them to the remote server located in the IoT cloud via the collocated LoRa-IoT gateway. Users can access these data anytime, anywhere, using a smart phone application. Additionally, users can obtain data locally from the concentrator when the WSN has limited or no internet access to ensure continuous data transmission to the IoT server.

**Figure 3:**
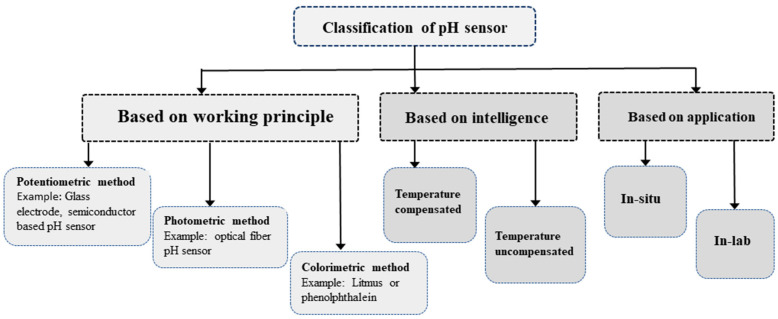
Classification of pH sensors based on three different perspectives.

The pH level measurement in aqueous media can be categorized in various criteria, such as intelligence, application, and operating principles as illustrated in Figure 3.

Based on its construction and operating principle, the pH-sensing method can be divided into three groups: potentiometric, photometric, and colorimetric. The most widely used pH sensors are based on potentiometric methods specifically using the glass electrode pH method [6]. This type of pH sensor uses a glass electrode filled with a Potassium Chloride (KCl) solution of a known pH and a reference electrode. However, the reference electrode is susceptible to corrosion during oxidation, leading to a decline in accuracy over time. Consequently, despite its popularity, this method presents challenges related to the need for frequent calibration. Electrochemical potentiometric pH sensors use two electrodes to directly monitor electrochemical reactions in order to determine the pH of biological samples [7]. The photometric method of measuring pH relies on expensive and complex glass or plastic optical fiber [8] to stimulate the compounds in the pH sensor and detect light emission. Conversely, the colorimetric method employs budget-friendly indicator dyes such as litmus or phenolphthalein, visually altering the color to indicate different pH levels without relying on any mathematical equation [9]. Nevertheless, this approach proves its limitations for in situ and real-time pH monitoring applications such as aquaculture.

There are two types of pH sensing based on the application: in situ and in-lab. While in-lab sensors provide more accurate results compared to their in situ counterparts, in situ sensors are better suited for real-time monitoring of aquaculture systems. Intelligent sensor nodes integrate temperature-compensation algorithm making it better suited for Biofloc application. Conversely, the uncompensated sensors involve inaccuracy in pH values with the fluctuations of temperature [10].

These available sensors pose challenges when deployed in aquaculture settings, attributed to their extended response times, restricted sensitivity, and stability issues. Furthermore, their design hinders seamless integration with other devices such as with wireless sensor nodes. These limitations underscore the need for the development of sensors that align with the required criteria.

The primary contribution of this work is the development of an affordable, temperature-compensated pH sensor to enable electronic calibration and in situ application for real-time monitoring of water quality in Biofloc environments. In our approach, multiple sensors are integrated into a local WSN through LoRa. This facilitates instantaneous data retrieval for effective management and ensures long-term data availability for future research, as well as comprehensive analysis of Biofloc environment parameters, including yield and quality.

To enhance the sensor’s accuracy and stability, a lightweight Machine Learning (ML) algorithm has been incorporated at the sensor node. This algorithm serves the dual purpose of temperature compensation and multipoint data compression. The strategic use of LoRa technology and the lightweight ML algorithm is focused on achieving low energy consumption within the local WSN, thereby extending the overall lifespan.

## 2. Related Works

The recent literature related to the development of pH sensors and their applications in a WSN is reviewed. Development of electrode-based sensors is found suitable for wireless sensing applications aided by various compensation and compression algorithms.

Yuqi Chen and Richard Compton in [11] have developed a bespoke calibration-free pH sensor using an in situ modified iridium electrode for applications in seawater. The electrochemical behavior of an iridium wire in air-saturated synthetic seawater is investigated, and the formation of pH-sensitive surface layers is observed. However, they do not concentrate on temperature compensation of pH measurement.

Riedel et al. in [12] demonstrate an ISFET-based agricultural sensing system for water and soil, focusing mainly on drift compensation and offset correction mechanisms. However, they do not consider enough the response time, sensitivity, flexibility of sensing elements, and disposal options.

Zhang et al. in [13] demonstrate a miniaturized pH sensor utilizing an assembly-free ball lens on a tapered multimode optical fiber (TMMF), offering the potential to revolutionize pH sensing in biochemical applications. They provide improved signal collection efficiency, which tackles the ongoing problems caused by low signal-to-noise ratios. Though effective, it’s a costly solution for aquaculture applications.

Swelling action of the polyvinyl alcohol (PVA)/polyacrylic acid (PAA) hydrogen sensing sheet is used in [14] to generate optical path modulation at different pH solutions. In addition to the complex structure, this photometric pH sensor is not suitable for integration in a wireless sensor node for real-time applications.

Wang et al. in [15] have developed a micro electro mechanical system (MEMS) of three electrodes to measure the temperature and pH focusing on better temperature compensation. Machine Learning (ML) algorithms were utilized to overcome issues related to the size of the electrode and stability at high temperature. However, they do not concentrate on issues like corrosion of the electrode due to the chemical reactions with water-based substances.

## 3. Technical Background

### 3.1. pH Measurement

Various methods are employed for pH measurement depending on the application. The colorimetric approach involves immersing an indicator in the test solution, and the resulting color change is matched to a specific color representing a particular pH. In the photometric method, a photodiode and LED detect luminescence in the pH sensor by stimulating its components. However, both colorimetric and photometric methods have limited applications due to their structural constraints and lower accuracy. Among the electrode-based potentiometric pH measurements, the glass electrode method, using a glass electrode and a reference electrode, measures the voltage between the two electrodes to determine solution pH. The antimony electrode method uses a polished antimony rod to gauge pH by comparing potential differences with a reference electrode.

The semiconductor electrode method utilizes a semiconductor device to measure ion concentration fluctuations in a solution, detecting changes in current flowing through the transistor. Platinum black is applied to the platinum wire or plate to create a hydrogen electrode, immersed in the test solution, and charged with electricity until saturated with hydrogen gas. The potential difference between the platinum black electrode and the silver chloride electrode is then measured to get the pH.

### 3.2. Temperature Compensation of pH

The Nernst equation establishes a connection between pH and temperature [16]. In a solution, the concentration of hydrogen ions can change with the temperature fluctuations. Table 1 illustrates the impact of temperature increases on pH values [17]. Specifically, the pH value increases for acidic solutions when the temperature increases. Conversely, for neutral or alkaline solutions, the pH value decreases as the temperature increases. To ensure accurate readings, it is essential to correct the pH values for temperature variations. Temperature compensation is employed to adjust the pH at the measurement temperature to the pH at a reference temperature of 25 °C.

### 3.3. Biofloc Fish Farming

Biofloc is an eco-friendly fish farming technique designed to minimize environmental impact, founded on the principles of in situ microbial growth and flocculation. This system ensures water quality by maintaining a higher Carbon-Nitrogen (C-N) ratio through additional carbohydrate supply. The composition of Biofloc includes microorganisms like bacteria, algae, fungi, invertebrates, and detritus. The toxic nitrogen species immobilize more rapidly in Biofloc making water quality preservation a key aspect.

Table 2 summarizes the specifications of a Biofloc system. A Biofloc farm typically comprises multiple tanks, each 4 m in diameter, 1.5 m in height, and 1.2 m in depth, with a storage capacity of about 15,000 L of water per tank. Key water quality characteristics include pH within the range of 6.5 to 8.5, alkalinity, dissolved oxygen (DO) at 5 mg/L, temperature, total dissolved solids (TDS) at 600 ppm, ammonia at 0.5 ppm, and nitrate at 150 ppm. Biofloc systems are versatile and suitable for various types of aquatic life including: air-breathing fish such as Singhi, Magur, Pabda; those that do not breathe air like Common Carp, Rohu, and Milkfish; as well as shellfish like Vannamei and Tiger Shrimp [18].

### 3.4. ML for Normalization, Calibration and Compression

ML algorithms are employed for multipoint data compression, data normalization, and temperature compensation. This involves compressing multipoint data into single data points. Subsequently, the compressed dataset undergoes normalization to identify outliers and assess data quality. The processed dataset is then obtained by verifying the remedial measures applied to the normalized data. Once the dataset of predictor variables is selected from the entire dataset, the statistical analysis and residuals of the model are examined and fitted. Following further confirmation of corrective measures, a provisional final model is chosen. Subsequently, the model’s validity is assessed, and a predicted output dataset is generated.

### 3.5. LoRa for WSN

LoRa is a low-power wide-area wireless network (LPWAN) technology that is increasingly used in wireless sensor networks (WSN). Data gathered from wireless sensors are wirelessly transmitted over LoRa to the IoT gateway and then to Cloud storage or a local server. LoRa has long-range and low energy consumption, which helps to increase WSN coverage and energy efficiency [19]. Therefore, use of LoRa in aquaculture applications will extend the overall system lifespan of sensor nodes powered by battery [20]. In Table 3 other parameters of various LPWAN technologies (LoRa, Sigfox, NB-IoT) are compared. The aforementioned parameters make it evident that NB-IOT’s high energy consumption makes it unsuitable for aquaculture applications. Considering bandwidth and data rate, LoRa is the optimal solution for aquaculture applications [21] over Sigfox.

## 4. Proposed Solution

To develop a low-cost, multi-point pH sensor setup for a highly dynamic Biofloc environment, several challenges have been addressed. This section explains how we approach certain problems and come up with solutions.

### 4.1. Features of Proposed Solution

The main focus of this research is to develop a new pH sensor with temperature compensation and simplicity to integrate with an IoT sensor node for in situ applications.

**Figure 4:**
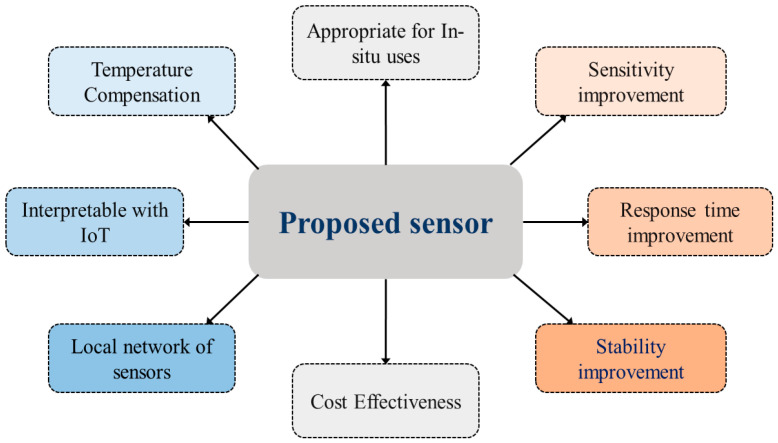
Features of proposed Solutions.

Other features as highlighted in Figure 4, such as sensitivity, response time, stability, and low cost are also taken into consideration. We have also concentrated on developing a local network of sensors at multiple depths. The Local network has a concentrator that collects data from the sensors wirelessly and transmits to the IoT server while acting as the LoRa-IoT gateway. 

### 4.2. Construction of pH Sensor

#### 4.2.1. Construction of Electrode

Electrodes of a pH sensor are used to measure the generated voltage in a solution due to the change in Hydrogen ion (H+) concentration. In the proposed pH sensor, a biasing resistor replaces the reference electrode as used in glass-electrode system. Hence, the current in a pH neutral solution is determined by the resistor instead of a chemical of known pH. Among the various types of electrodes [22] a good sensing electrode has qualities including robustness, smoothness, high resistance, chemical neutrality, response time, durability, and temperature adjustment [23]. The selection of a suitable material, length, and relative position of the electrodes are important to ensure its reliability.

We study the materials used as electrodes in commercially available pH sensors, including silver, silver chloride, platinum, chromium, and stainless steel. Characteristics as a pH sensing electrode such as biocompatibility, resistivity, corrosion resistance, electrode potential, and chemical reactions [24,25] led us to select the materials.

Table 4 highlights the characteristics of gold (Au), silver (Ag), and chromium (Cr) as the shortlisted materials. Gold is found least active with other substances in water to generate oxides and is more durable, corrosion-resistant, and biocompatible than the other two materials. When used as a pH electrode, the flat surface of gold lowers the adherence of organic particles and microbes, reducing biofouling. This ensures consistent performance in biofouling-prone situations as well as making cleaning and maintenance easier. One major benefit of gold electrodes in saline environments is their great resistance to corrosion caused by chloride and their overall stability over a broad range of salinities. Long-term electrolysis was avoided by using a bipolar biasing current of the electrode. Gold-based electrodes can offer a long-lasting and efficient solution for dynamic aquaculture conditions by taking these considerations into account.

**Figure 5:**
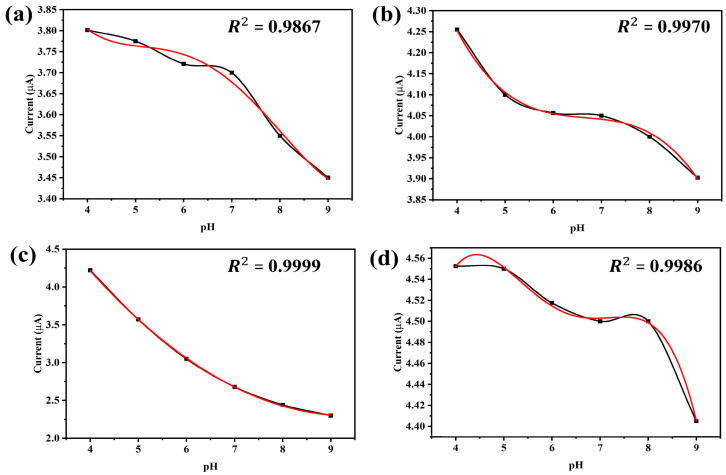
Behavior of the probe at different lengths. (**a**) Current response of pH sensor when probe length is 6 mm. (**b**) Relation between current and pH when probe length is 9.5 mm. (**c**) Current response of the sensor when probe length is 8 mm. (**d**) Current and pH relation with 10 mm probe length.

Potential differences due to Hydrogen ion concentration between two electrodes made of these solutions. The potential difference is measured in mV scale and it is found decreasing below 0 V with the increase of pH and increasing above 0 V with the decreasing of pH. We found gold (Au) has a superior relationship between pH value and potential value compared to chromium (Cr) and silver (Ag). The pH potential depends on the surface area of the electrodes, therefore, different shapes, dimension and placement of the electrodes are monitored to get the optimum sensitivity. Figure 5 shows the sensitivity of the gold-plated electrode for different lengths (6 mm, 9.5 mm, 8 mm and 10 mm) at different pH values. The trend-line reliability (*R^2^*) is shown for different polynomials to find out the best pH to current relationship. Figure 5c depicts that the highest (*R^2^* = 0.9999) reliability is obtained for second order polynomial using the 8 mm long electrode. The 9.5 mm long electrode gives a similar (*R^2^* = 0.997) result for 3rd order polynomial. The longest (10 mm) and shortest (6 mm) are shown as less reliable even for 3rd or higher order polynomials. Based on the relations between current response and pH values, we selected an 8 mm long electrode as the pH sensor.

#### 4.2.2. Biasing for pH-Neutral Point

**Figure 6:**
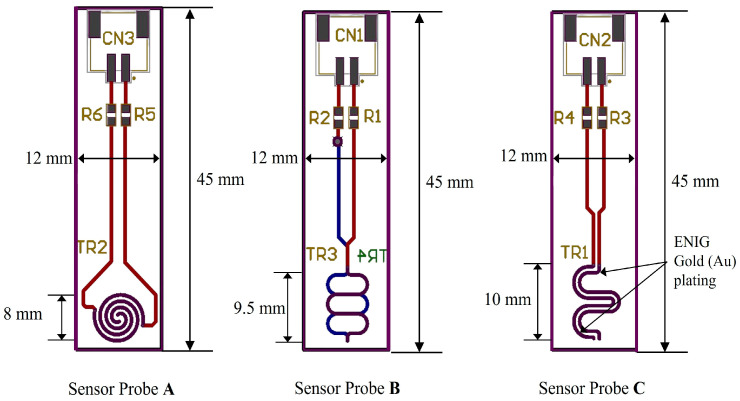
Pattern and length variants of sensor electrodes. Sensor probe A has 8 mm round shape gold plating; B has 9.5 mm and C has 10 mm gold plating.

To address calibration challenges in the glass-electrode setup, we opt for electronic biasing of the probe. Initially, we measure the current using a multimeter (Fluke 17B+, Fluke, Washington, DC, USA) of a chemically biased probe in a pH 7 reference solution, yielding 723 μA using reference electrode method. To achieve the same current across our probe, we introduce a current limiting resistor to one of the electrodes. Using the same resistor, we measure currents across pH solutions ranging from 4 to 9, recalculating electrode linearity in a similar way to determining electrode length as shown in Figure 5. After testing with a ±30% variation in resistor values, we settle on an optimal value of 33 kΩ. Figure 6 illustrates the electrode wiring diagram, including the current limiting resistor, during current measurement to determine the best bias position and resistor value.

### 4.3. Working Principle of the Sensor

pH denotes the concentration of hydrogen ions in a solution, represented logarithmically [26]. It is defined by Equation (1):

(1) $$\;pH=-log\;[\;H^{+}]\;\;\;$$

Here, [*H*^+^] is the equilibrium molar concentration of hydrogen ions (mol/L) in the solution. The Nernst equation, shown in Equation (2), calculates the concentration of hydrogen ions (*H*^+^) and determines the potential difference between two electrodes in the potentiometric pH measurement method [27]:

(2) $$E=\frac{RT}{nF}\times log\frac{C1}{C2}$$

where, *E* = potential difference in mV, *R* = universal gas constant, *F* = Faraday constant, *T* = absolute temperature in kelvin (K), *n* = number of charges of the measured ion, *C*1 = active ion concentration of reference solution, *C*2 = active ion concentration of the solution of unknown pH.

The Nernst equation illustrates an inverse relationship between the voltage produced and pH. To elaborate, the detected voltage increases as pH decreases below 7, and decreases as pH exceeds 7.

### 4.4. Lab Setup of the Sensor

**Figure 7:**
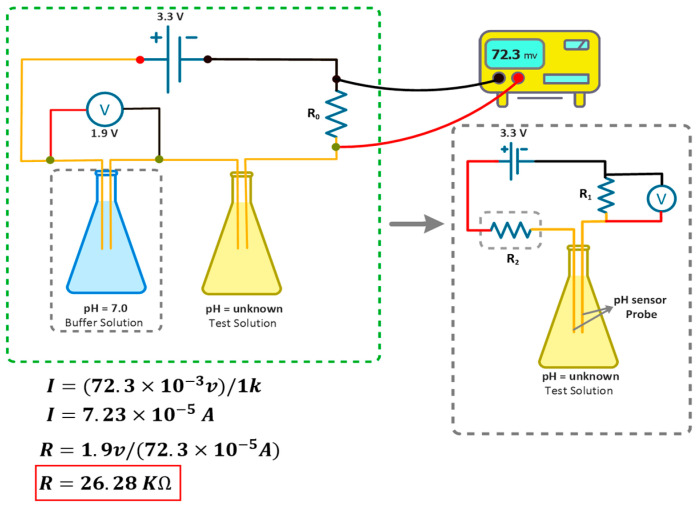
Working principle of the sensor node.

To develop a fully electrical pH sensor suitable for IoT aquaculture applications without reliance on chemical solutions, we replace the reference electrode typically found in electrode-based pH sensors. As depicted in Figure 7.

Our sensor’s electronic circuit incorporates a resistor in lieu of the traditional reference electrode. This approach utilizes a current limiter to measure pH, eliminating the need for chemical components in our sensor setup.

**Figure 8:**
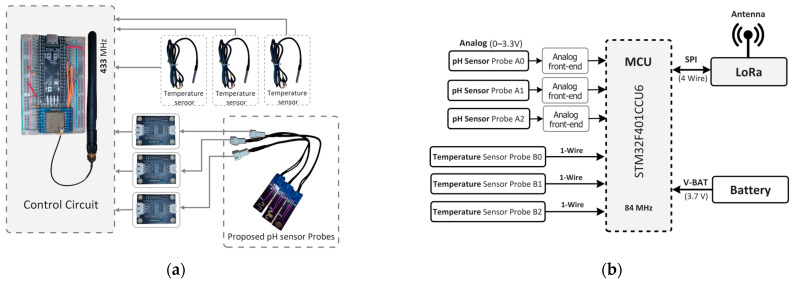
The lab setup of the sensor. (**a**) Hardware block diagram of the pH sensor with control circuit. (**b**) Schematic diagram of the sensor.

In Figure 8a, our sensor node is depicted with a hardware block diagram showcasing our proposed pH sensor probe and pH sensing circuit. The sensor node comprises a microcontroller (STM32F401, STMicroelectronics, Geneva, Switzerland), a LoRa module with 433 MHz (SX1278, Semtech, Camarillo, CA, USA) for IoT connectivity, and a temperature sensor (DS18B20, Analog Devices Inc., Wilmington, MA, USA) for temperature compensation. The proposed pH sensor is developed on a printed circuit board (pHSensorV1.0, JLCPCB, Hong Kong, China) with a gold-plated finish using 2U” Electroless Nickel Immersion Gold (ENIG) surface treatment, optimizing trace width to 0.5 mm for enhanced conductivity. Sized at 40 × 10 mm, the PCB includes a length of 8 mm.

ENIG-plate dedicated to collecting data from pH water solutions. Integration with the main MCU is facilitated through an analog frontend that measures the current, converts it to voltage and amplifies it for the inbuilt ADC of the MCU.

Figure 8b illustrates the schematic of our sensor node, detailing three ADC pins utilized for the pH sensor probe (A, B, C) and three digital pins employed for temperature sensors to gather data across various pH solutions. Temperature sensing utilizes the One Wire Communication Protocol (OWCP), while the LoRa module communicates via a 4-wire Serial Peripheral Interface (SPI) bus.

### 4.5. Data Collection and Processing

**Figure 9:**
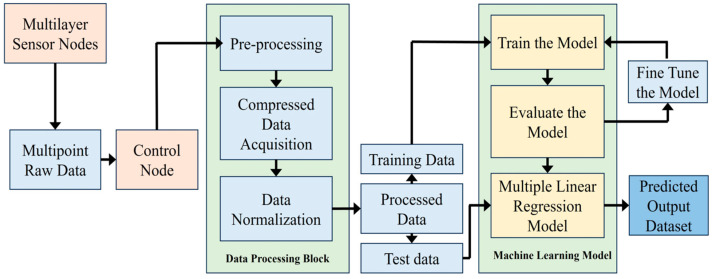
Data collection and processing block diagram.

In the proposed system, multiple sensor nodes gather pH data from the Biofloc tank. At the local concentrator (also acts as the local control node), these raw data undergo preprocessing, compression, and normalization to ensure accuracy. Subsequently, the data is categorized into training and test sets for training a linear regression model. After training, the model undergoes evaluation and fine-tuning to enhance performance. Once optimized, the model can predict pH values within the tank. The process is visualized in Figure 9.

### 4.6. Overview of System Architecture

**Figure 10:**
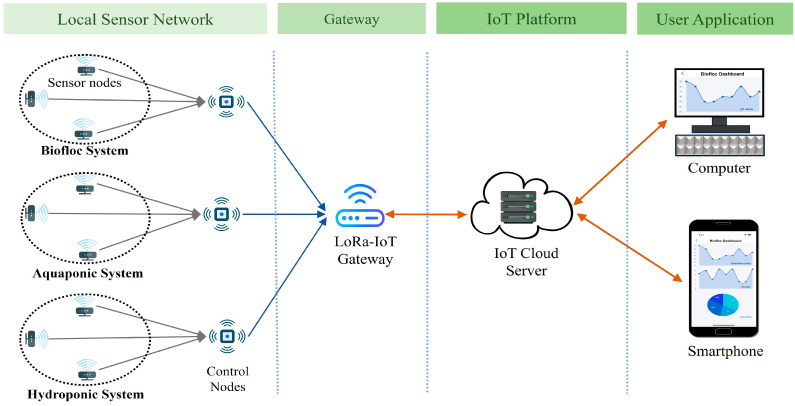
System Architecture of proposed solution where multiple sensor nodes are placed in different aquaculture systems. Control node collects sensor data and store the data to an IoT platform through a gateway.

For IoT-based in situ monitoring of aquaculture, a control node along with multiple sensor nodes is used to establish a local network of sensors in a Biofloc tank. To obtain accurate data in the noisy environment of the Biofloc tank, local sensor nodes are positioned at various depths within the tank. Figure 10 demonstrates the system architecture utilized in our investigation. The control sensor node in the local network collects pH data from the local sensor nodes and transmits it via the LoRa-IoT gateway. A LoRa-IoT gateway in a cloud network is used for transmitting sensor data to the cloud server. Biofloc users access the application layer through their computer or smartphone application.

## 5. Performance Analysis

For IoT-based in situ monitoring of aquaculture, the proposed system employs a control node and multiple sensor nodes to establish a local network within the Biofloc operation area. Sensor nodes are strategically positioned at various depths to ensure accurate data collection in the midst of noisy environment of the tank. Figure 10 illustrates the proposed smart Biofloc architecture. The control sensor node within the local network gathers pH data from these sensors and transmits it via a LoRa-IoT gateway. The LoRa-IoT gateway functionality in the local control node forwards this data to a cloud server. Biofloc users can access the application layer through their computers or a smartphone application.

### 5.1. Current Response of pH Sensor

**Figure 11:**
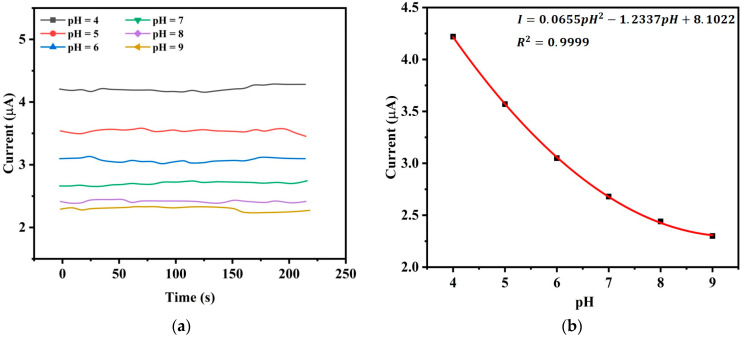
Relationship between current and pH. (**a**) Current response of the pH sensor at different pH values. (**b**) Current and pH relation with high linearity.

The current response of pH sensor indicates how the current varies when the pH of a solution changes [28]. Figure 11a demonstrates how the current changes over time for a given pH value which is chosen from pH 4 to pH 9 with an increment of pH 1. Here, we consider pH values between 4 and 9 since aquaculture applications are well suited to this range. The measuring temperature is 25 °C. When the pH is high, we observe that the electrode’s current decreases and for a given pH, the current remains to be stable to a fixed value with a minor variation over time. Figure 11b is obtained from Figure 11a by averaging all the current values at given pH where we observe an inverse nonlinear relationship between the current and pH. The best fit second-order polynomial that relates current to pH is found to be

(3) $$I=0.0655pH^{2}-1.2337pH+8.1022$$

with $R^{2}=0.9999\;$ that shows a strong correlation.

### 5.2. Temperature Compensation with ML Agorithm

To develop a relationship between pH, temperature, and current, multiple linear regression is employed using TensorFlow. To verify the created regression model has the minimum sum of squared errors, the multiple linear regression analysis is approximated using the least squares approach [29]. A dataset is collected that has one dependent variable (pH) and two independent variables (temperature and current). The dataset is divided into two categories: testing and training. The training dataset is fitted to a multiple linear regression model. The test dataset is used to make predictions. Using the machine learning model, the following functional relation can be developed between the solution’s pH, temperature (T), and current (I)

(4) $$\;pH=K_{0}+K_{1}T+K_{2}I\;$$

where, Temperature in °C and *I* = Current value in micro-Ampere (μA). Metrics such as the coefficient of determination ($R^{2}$) value and squared error (SE) are used to assess the model. The output specifications of the multiple linear regression model, provides the following values of the coefficients.

$${\;K}_{0}=10.376$$

$$K_{1}=0.0637$$

$$K_{2}=-0.1858$$

Table 5 presents a sample of the training data out of the total of 2924 observations for the regression model consisting of current values observed at given pH and temperature of reference solution. Using a separate set of test data of 1235 observations, the regression model achieves standard error = 0.3 and the *R^2^* value = 0.94. This performance is reasonably accurate for the application of pH measurements in a dynamic Biofloc environment.

**Figure 12:**
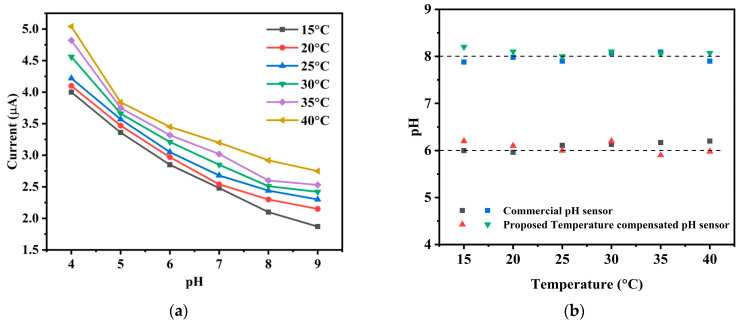
Temperature compensation of the pH sensor. (**a**) Different pH values at different temperature without temperature compensation (**b**) Measured pH with respect to temperature using our temperature compensated pH sensor and a commercial pH meter.

The Figure 12a demonstrates how the current response of the pH sensor changes due to change in temperature. When the temperature increases with an increment of 5 °C, current response shifts upward which captures the temperature effect in pH measurement causing errors in the pH readings without any temperature compensation. In aquaculture environments, where temperature fluctuations are common, the model continuously monitors and compensates for these changes, ensuring long-term accuracy. In Figure 12b the obtained values of pH 6 and 8 at different temperature with our temperature compensated sensor are compared with the values of a commercial pH meter. Figure 12b also shows how the output reading of the proposed pH meter continuously indicates pH 6 as a result of the adjustment when the temperature varies. Taking temperature variations into account, this finding suggests that, in comparison to its commercially available counterpart, the proposed novel low-cost designed sensor with temperature compensation offers comparable accuracy. Therefore, we can claim that the temperature compensated sensor provides good accuracy and efficient temperature adjustment at a commercial level resulting in the decreased variance in pH measurements. This characteristic is important in various applications where precise pH measurement is essential, ensuring that the sensor’s performance is reliable even under varying temperature conditions.

### 5.3. Response Time

**Figure 13:**
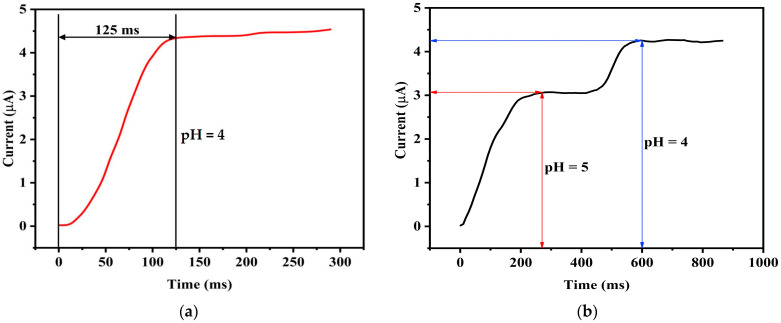
Response time of the sensor. (**a**) Response time of the pH sensor when pH is 4 (**b**) Dynamic response time with alternation of pH.

One of the important quality indicators of a pH sensor is the response time. To evaluate the response time of our pH sensor, a pH 4 solution is considered for testing at a controlled temperature of 25 °C. The resultant current-time variation is illustrated in Figure 13a, where it is evident that the sensor takes approximately 125 ms to reach 90% of its steady-state reading. Subsequently, as shown in Figure 13b, a different experimental condition involving a change in the pH of the solution from pH 5 to pH 4, the dynamic response time of the sensor is examined. Here, a dynamic response time of 320 ms is observed. This finding underscores the sensor’s ability to swiftly and accurately adapt to variations in pH, making it a suitable tool for real-time monitoring and control applications. These results affirm the effectiveness and efficiency of the proposed pH sensor in responding to dynamic changes in pH levels.

The sensor’s response time ensures consistent performance across a range of conditions. The robust construction and optimized materials of the sensor contribute to its ability to deliver accurate and timely measurements, making it well suited for dynamic environments.

### 5.4. Sensitivity

**Figure 14:**
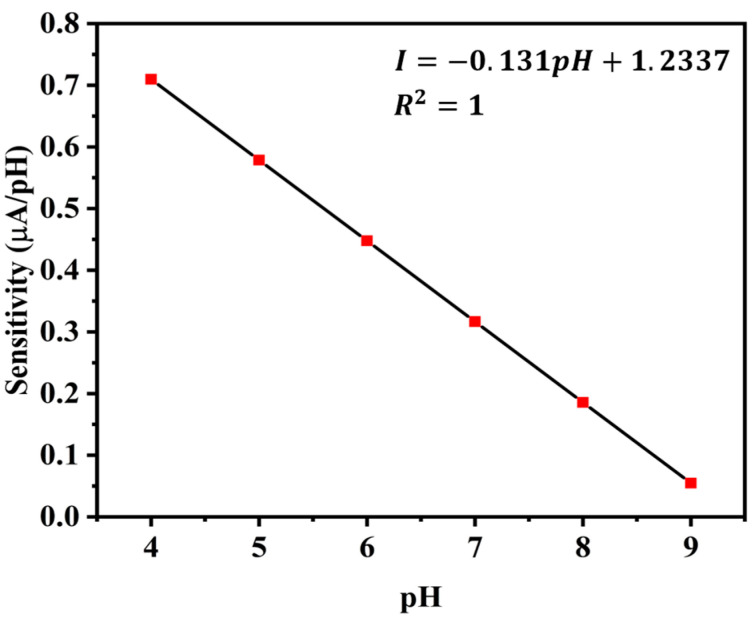
Sensitivity of the sensor for different pH values at 25 °C.

Another performance indicator is the pH sensor’s sensitivity, or the slope of its current response curve [30]. The current response of this sensor is displayed as a second-order non-linear equation in Figure 11b. To obtain the sensitivity, we therefore take the derivative of that equation. At a constant temperature of 25 °C, we obtain different sensitivities for different pH values. The sensitivity values in μA/pH for various pH solutions are displayed in Figure 14.

pH and sensitivity have a linear relationship with a negative correlation. Thus, the following formula can be used to determine the sensor’s sensitivity.

(5) $$\;I=-\left(m\times pH\right)+C$$

where, ***I*** = sensitivity in μA/pH,
***m*** = slope of the straight line,
***C*** = constant as y-intercept
The values of ***m*** and ***C***, as determined by curve fitting, are ***m* = −0.131** and ***C* = 1.2337.**

ISFET and ZnO sensors offer higher sensitivity while they are significantly more expensive to produce and maintain [31]. The proposed sensor strikes a balance between improved sensitivity and cost-effectiveness, making it a more practical and accessible solution for applications like aquaculture, where affordability and reliability are critical considerations.

The trade-offs between sensor sensitivity and long-term stability are a key consideration in designing pH sensors for aquaculture monitoring. High sensitivity is crucial for detecting subtle changes in water quality, while long-term stability ensures reliable operation with minimal maintenance. By optimizing material selection and incorporating protective measures, a balance is achieved that meets the practical demands of aquaculture environments.

### 5.5. Stability

Stability is another performance parameter that determines output precision. Here, we would like to assess the long-term stability and performance of a pH sensor, a factor that reflects the reliability of pH measurements. The use of bipolar biasing current through the electrode will make it more tolerable to the electrolysis/electroplating effect, hence maintaining physical properties unchanged for a long time, however, regular cleaning is still recommended to counteract the effects of dissolved organic matter. Regular cleaning, anti-fouling coatings, or the use of mechanical wipers can help maintain electrode performance. Gold’s smooth surface also makes it easier to clean compared to rougher materials.

Robust materials like gold, along with bipolar biasing current is resist corrosion and fouling. Designing electrodes with smooth surfaces minimizes fouling sites. Use wipers, brushes, or ultrasonic cleaning systems to periodically remove fouling agents from the electrode surface.

A conventional reference electrode relies on an internal electrolyte (KCl) and a redox couple (Ag/AgCl), both of which can become contaminated or depleted over time, resulting in instability. Long-term stability is improved by removing the necessity for the chemical system by incorporating the buffer solution’s electrochemical analogy to a known current-limiting resistor. Additionally, reference electrodes are fragile and prone to damage in harsh environments like aquaculture, whereas a biasing resistor is more robust and less likely to degrade, further enhancing stability.

Due to changes in the electrolyte concentration or junction blockage, the voltage at the liquid junction of conventional reference electrodes can fluctuate. A biasing resistor, which does not require a liquid junction, can reduce the fluctuation. By substituting a biasing resistor for a traditional reference electrode, drifts caused by the mechanical and chemical constraints of the reference electrode can be minimized and long-term stability enhanced.

**Figure 15:**
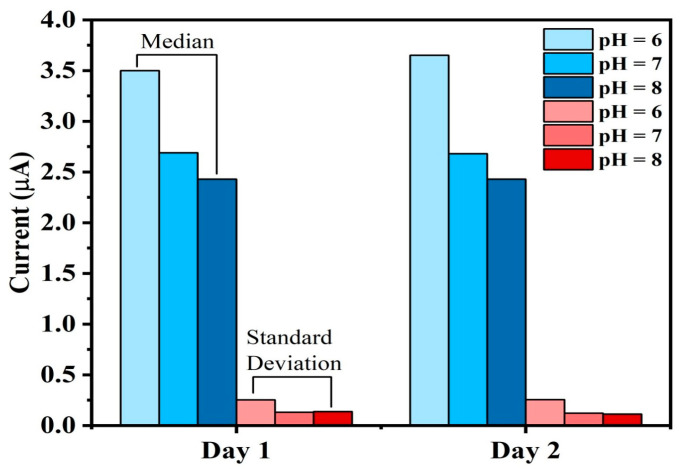
Stability of the sensor; Measured median and standard deviation values of pH 6,7 and 8 over two days.

To capture the notion of stability, a meticulous and extended observation is conducted, involving the measurement of pH values across three distinct solutions, each with pH values of 6, 7, and 8. These measurements were consistently recorded over two days. This extended observation period is essential for validating the sensor’s consistent performance over time. Figure 15 illustrates the outcome of this analysis based on two days of observations. The median and standard deviation of current values of pH 6, 7, and 8 are measured based on the data taken over each day to check the stability. It is evident that the median and the standard deviation of the current values remains to be the same for each of the observed days. This observation provides the evidence that the sensor maintains a consistent level of performance throughout the experiment, ensuring stable and reliable pH measurements over time.

The stability tests were conducted for pH 6, 7, and 8 because these values represent the optimal and most relevant pH range for a Biofloc aquaculture environment. In Biofloc systems, maintaining pH within this range is critical for the health and survival of fish, as extreme pH conditions (such as pH 4 or 9) can cause stress or even death to aquatic organisms. Since the sensor is designed for practical aquaculture applications, its performance was validated under conditions that reflect real-world operational requirements. Testing within the pH 6–8 range ensures the sensor’s reliability and accuracy in environments where maintaining stable water quality is essential for fish health and system productivity. Extreme pH conditions, while theoretically important, are not typically encountered in well-managed Biofloc systems, making the chosen range both practical and scientifically justified.

### 5.6. Comparative Evaluation

This subsection compares the sensor properties and performance parameters of our study with the works mentioned in the related work section. Table 6 presents a comparison of the significant features of all the works discussed. Although our work is based on in situ application, we also consider in-lab, online or remote monitoring of pH for a wide range of comparisons, summarized in this table. For most of the works, accuracy is not quantified clearly. The measurable pH range of our sensor spans from pH 4 to pH 9 (pH buffer solution, Zhengzhou Wollen Instrument Equipment Co., Ltd., Zhengzhou, China), which aligns with the optimal pH range for Biofloc fish farming. Our work is based on the electrical current limiter approach which is simple and easy to use compared to MEMS, ISFET, optical fiber electrode or any other electrode types. Compared to all other works our sensor’s response time is improved by 4 times. Sensitivity is also improved significantly. There is limited information available in the literature regarding the actual cost management of existing sensors. However, we have provided a comparison of the technologies used, which offers insights into the potential costs of the sensors listed in the comparison table, relative to the proposed sensor. Furthermore, we conducted an analysis of industrial-level pH meters and found that temperature-compensated pH meters are generally more expensive [32]. In contrast, the calibration-free design and simple construction of the proposed pH sensor make it a more affordable and cost-effective solution for aquaculture applications. Table 7 presents a comparative view of the limitations of existing pH sensors along with the solutions of the proposed work.

## 6. Conclusions

A low-cost, temperature-compensated electronic calibration-based pH sensor for in situ aquaculture applications was presented in this paper. Temperature compensation was achieved by a machine learning algorithm to eliminate temperature-related errors. Electronic calibration is achieved by applying a current limiting approach to the sensor which eliminates maintenance and calibration problems of the pH sensor. A full architecture of how a user can remotely access the pH data of a local sensor network is also proposed. The proposed designed sensor demonstrates a reaction time of 125 ms and sensitivity of 0.316 µA/pH with a satisfactory stability. Therefore, the outcomes of these performance parameters ensure the suitability of this sensor for real time monitoring of aquaculture applications. In the long term, we intend to develop a multi-sensor system for complete monitoring of other water quality parameters of a Biofloc tank. Enabling compensation for nonlinearities in sensor response across the Biofloc tank with various chemical and biological compositions is another long-term objective.

## Figures and Tables

**Table 1:** Temperature to pH relationships.

Temperature (°C)	pH 4.01	pH 7	pH 10.01
0	4.01	7.12	10.32
10	4	7.06	10.18
20	4	7.02	10.06
25	4	7.01	10.02
30	4.01	6.98	9.97
40	4.03	6.97	9.89
50	4.06	6.97	9.83

**Table 2:** Specifications of a Biofloc a system.

Parameters	Specifications
Tank size	Diameter 4 m; Height 1.5 m; Water depth 1.20 m
Water holding capacity	15,000 L/tank
Water quality parameters	pH, Alkalinity, Dissolved Oxygen, Temperature, TDS, Floc density-25–40 mg/L, Ammonia, Nitrite, Nitrate
Survival (%)	80
Feeding frequency	early stage 4 times per day, later 2 times per day
Types of species	Air breathing, Non air-breathing, Shellfishes

**Table 3:** Comparison among different LPWAN technologies.

Specifications	LoRa	Sigfox	NB-IoT
Range	5 km (urban), 20 km (rural)	10 km (urban), 40 km (rural)	1 km (urban), 10 km (rural)
Energy consumption	0.03/message	Not available	0.13/message
Bandwidth	250 kHz and 125 kHz	100 Hz	200 kHz
Maximum data rate	50 kbps	100 bps	200 kbps
Maximum messages/day	Unlimited	140 (UL), 4 (DL)	Unlimited
Adaptive data rate	Yes	No	No
Allow private network	Yes	No	No

**Table 4:** Comparison of selected materials.

Material	Durability	Oxide Formation	Corrosion Resistance	pH and mV Relation	Biocompatibility
Gold (Au)	Very long	No	Very high	Best	Superior
Silver (Ag)	Long	Sometimes	Quite resistive	Better	Quite good
Chromium (Cr)	Less long	Unstable in oxygen	Good	Good	Not that good

**Table 5:** Training data of multiple linear regression model.

pH	Temperature (°C)	Current (μA)
4	18.97	4.04
4	20.00	4.09
4	24.03	4.20
4	28.49	4.35
5	20.16	3.45
5	24.02	3.54
5	29.50	3.61
6	22.60	3.06
6	27.10	3.11
6	35.71	3.35
6	38.88	3.53
7	18.02	2.48
7	22.46	2.60
7	25.81	2.70
7	30.03	2.84
8	16.50	2.11
8	23.70	2.42
8	32.28	2.65
9	20.00	2.44
9	36.80	2.62

**Table 6:** Output comparison among proposed work and references.

Features	Proposed Work	[15]	[13]	[12]	[11]
Monitoring Type	In situ	In situ	Not available	Real time	In situ
Uses	Biofloc aquaculture	Drinking water	Biochemical applications	Agricultural applications	Sea water monitoring
Accuracy	Good	Good	Good	Good	Good
pH range	4–9	Not available	6–8	Not available	0–14
Technology/Process	Electrical current limiter	MEMS	Tapered Multimode Optical Fiber	ISFET based sensing	Metal oxide micro electrode
Response Time	125 ms	<4 s	0.5 s	Not available	Not available
Sensitivity	0.316 μA/pH	0.288 mA/pH	1001.57 counts/pH	55 mv/pH	70.1 ± 1.4 mv/pH
Cost	Low	Not available	Not available	Not available	Not available
Power Consumption	Low	Not available	Low	Not available	Not available
Stability	High	High	High	Low	High

**Table 7:** Comparison among limitations of existing sensors and the solutions of proposed work.

Types of pH Sensors	Limitations of the Existing Sensors	Solutions of the Proposed Sensor
Traditional potentiometric pH sensors	Frequent calibrations and maintenance	No need for calibration and frequent maintenance
Different electrode material-based pH sensors	Inaccuracies due to biofouling and electrode corrosion	Resistive to biofouling and corrosion
Optical Fiber pH sensors	Sometimes cannot be implemented with IoT based systems for remote monitoring	Integrated with IoT based systems for remote monitoring and real time data collection
ISFET/IGFET based pH sensors	Need daily calibration, complex design and costly solutions for aquaculture	Simple construction with current limiting approach
Temperature uncompensated pH sensors	No compensation for temperature fluctuations	Temperature compensation reduces inaccuracies

## Data Availability

Data are contained within the article.
